# Changes in the Secretion of Anti-Inflammatory Cytokines and Acute-Phase Proteins in the Uterus after Artificial Insemination in the Mare

**DOI:** 10.3390/ani10122438

**Published:** 2020-12-19

**Authors:** Katarzyna Wojtysiak, Wojciech Ryszka, Tadeusz Stefaniak, Jarosław Król, Roland Kozdrowski

**Affiliations:** 1 Department of Reproduction and Clinic of Farm Animals, Faculty of Veterinary Medicine, Wroclaw University of Environmental and Life Sciences, Plac Grunwaldzki 49, 50-366 Wroclaw, Poland; katarzyna.wojtysiak@upwr.edu.pl; 2 Equi Salus, Glinno 65, 64-300 Nowy Tomyśl, Poland; w.ryszka@equisalus.pl; 3 Department of Immunology, Pathophysiology and Veterinary Preventive Medicine, Faculty of Veterinary Medicine, Wroclaw University of Environmental and Life Sciences, C.K. Norwida 31, 50-375 Wroclaw, Poland; tadeusz.stefaniak@upwr.edu.pl; 4 Department of Pathology, Faculty of Veterinary Medicine, Wroclaw University of Environmental and Life Sciences, C.K. Norwida 31, 50-375 Wroclaw, Poland; jaroslaw.krol@upwr.edu.pl; 5 Faculty of Biological and Veterinary Sciences, Nicolaus Copernicus University, Gagarina 11, 87-100 Toruń, Poland

**Keywords:** mare, artificial insemination, endometritis, cytokines, acute-phase proteins

## Abstract

**Simple Summary:**

Semen deposition into the uterus during mating or artificial insemination induces a rapid inflammatory response, and, in susceptible mares, persistent endometritis can develop. Cytokines are mediators involved in the regulation of the inflammatory process, and acute phase proteins are the most sensitive indicators of the inflammatory process. Therefore, the aim of this research was to determine the secretion of anti-inflammatory cytokines and acute-phase proteins in the uterus before and after artificial insemination in the mare. The obtained results indicate that the status of the mare before artificial insemination has little effect on the response measured shortly after artificial insemination. The presence of intrauterine fluid during estrus is not connected with the inflammation parameters investigated in this study at 7 h post artificial insemination. Detailed examination of the mare’s reproductive tract before and after artificial insemination guarantees high fertility.

**Abstract:**

The objective of the study was to evaluate the concentrations of interleukin-1 receptor antagonist (IL-1RA), interleukin-10 (IL-10), serum amyloid A (SAA) and haptoglobin (Hp) in uterine lavage fluid before and after artificial insemination (AI). Based on ultrasound examination, mares were divided into: Group 1 (*n* = 9), no fluid was detected in the uterus during estrus and 7 h after AI; Group 2 (*n* = 8), no fluid was detected in the uterus during estrus but 7 h after AI fluid was detected in the uterus; Group 3 (*n* = 8), fluid was detected in the uterus during estrus and also 7 h after AI. In all groups of mares, a significant increase in polymorphonuclear cells (PMN) and a significant increase in IL-1RA and SAA were recorded 7 h after AI. The obtained results show that, regardless of the status of the mare before AI, the endometrial response characterized by PMN influx, and SAA, Hp, IL-1RA and IL-10 production, is similar. The presence of intrauterine fluid during estrus is not connected with PMN influx but can impact uterine IL-1RA production at this time.

## 1. Introduction

Semen deposition into the uterus during mating or artificial insemination (AI) induces rapid chemotaxis of polymorphonuclear (PMN) cells into the endometrial tissue and uterine lumen [1]. This process is accompanied by lymphatic drainage and by uterine contractions. In mares resistant to persistent breeding-induced endometritis (PBIE), this process enables the clearing of the uterus from inflammatory fluid, bacteria and excess spermatozoa [2,3]. This process is also called a transient breeding-induced endometritis (TBIE) and occurs in each mare, and it should be considered an innate immune defense mechanism. It is important to resolve the TBIE before arrival of embryos into the uterus. In mares susceptible to PBIE, this process is disturbed and is manifested clinically by intrauterine fluid (IUF) accumulation. The presence of IUF 24 h and later after breeding is negatively correlated with fertility [1]. Unfortunately, the mechanisms underlying the progression of TBIE to PBIE are not fully understood [4]. Impaired uterine contractility is considered to be the most important reason for IUF retention [3,5]. Nitric oxide, a mediator of smooth muscle relaxation, probably plays an important role in the inhibition of uterine contractility in mares [6,7,8,9]. Risk factors associated with IUF accumulation in diestrus are as follows: age of the mare, parity, poor perineal conformation, infection of the uterus, uterine position within the abdominal cavity, abnormal cervical function, excess fluid retention during estrus, and uterine biopsy scores IIB and III (increasing of fibrotic and inflammatory changes in the endometrium) [1,3,5,10,11,12,13,14].

Another aspect that is not fully understood is the accumulation of IUF during estrus and its impact on fertility. This fluid can be anechogenic and is usually located in the uterine body [15]. Cytological examination in these cases does not confirm the presence of endometritis [15,16]. Nevertheless, the presence of IUF during estrus may suggest susceptibility to PBIE, especially if it exceeds a depth of 2 cm [10]. Additionally, the presence of IUF during estrus can be related to reduced pregnancy rates [17]. It was also shown that mares with IUF during estrus are more likely to have acute endometritis [18].

Cytokines are mediators involved in the regulation of the inflammatory process. They can be divided into two groups: (1) pro-inflammatory cytokines, for example interleukin-1β (IL-1β), interleukin-8 (IL-8) and tumor necrosis factor α (TNFα) which initiate inflammation, and (2) anti-inflammatory cytokines, for example, interleukin-1 receptor antagonist (IL-1RA) and interleukin-10 (IL-10) which inhibit pro-inflammatory cytokine production. Studies focused on the role of cytokines in the regulation of the inflammatory process in mares after challenge, based on the introduction into the uterus of dead spermatozoa [19,20,21,22] or after inoculation of the uterus with *Escherichia coli* [23], showed changes in endometrial mRNA expression for some cytokines, indicating their possible role in modulation of TBIE and its conversion into PBIE in susceptible mares.

Acute phase proteins (APPs), including serum amyloid A (SAA), which is the major APP and is recognized as the most sensitive indicator of the acute phase response in horses, and haptoglobin (Hp), which is a minor-to-moderate APP, were widely evaluated in healthy and sick horses [24,25]. Data concerning changes in the concentration of APPs in reproductive disorders in mares are not consistent. For example, the plasma SAA concentration in response to *E. coli* inoculation into the uterus appears to be dose dependent [23,26] and measuring APPs concentration is not a useful diagnostic tool for subclinical endometritis in mares [27]. However, in one study, elevated concentrations of APPs were found around the time of ovulation in mares in which early embryonic death was later detected [28]. It was also shown that APP concentrations are not influenced by AI in mares [4,29]. Generally, APPs are produced in the liver but Chapwanya et al. [30] showed that the endometrium of cows can also be a source of SAA. Previous studies showed the presence of SAA and Hp in fluid obtained from the uterus of cows suffering from subclinical endometritis or pyometra and from cows with healthy endometrium [31,32,33].

We hypothesized that: (1) AI induces production of APPs in the uterus; (2) the presence of uterine fluid during estrus influences the production of cytokines and APPs. Therefore, the objectives of the study were: (1) evaluation of the concentration of anti-inflammatory cytokines in uterine lavage fluid before and after AI and the determination of the role of chosen cytokines in the modulation of the inflammatory process associated with AI; (2) evaluation of the concentration of APPs (SAA and Hp) in uterine lavage fluid before and after AI.

## 2. Material and Methods

### 2.1. Animals

From a number of mares introduced into a commercial AI program during the 2016–2018 breeding seasons, 25 mares aged 4–20 years were selected for observation using the criteria described below. All procedures performed on these mares were approved by the owners and did not go beyond the routine veterinarian procedures performed on mares introduced to AI. The Local Ethics Committee in Wrocław, Poland, made a decision that these observations are out of the scope of the commission, while the guidelines contained in the directive 2010/63/EU of the European Parliament and of the Council of 22 September 2010 on the protection of animals used for scientific purposes were respected.

A detailed history was taken of each mare, and only clinically healthy mares without a history of reproductive disorders and with normal perineal conformation (over 80% of the labia length lay below the ischiadic arch of the pelvis) were enrolled into the study. For all examinations and procedures, the mares were restrained in an examination stock.

### 2.2. Ultrasound Examination

An ultrasound scanner and linear-probe 6 MHz (MyLab™One, Esaote, Italy) was used. Ultrasound examination of the reproductive tract was performed every day from early estrus and again 7h after AI. The diameter of the dominant follicle, endometrial edema and the presence and depth of IUF was recorded. Moreover, 14–16 and 40 days after ovulation additional ultrasound examinations were done for pregnancy diagnosis.

### 2.3. Experimental Groups

This study was based on ultrasound examination of the reproductive tract performed during estrus (endometrial edema and a dominant follicle were detected), and 7 h after AI the mares were divided into three groups:

1. Group 1 served as a control group. In this group, no fluid was detected in the uterus during estrus or 7 h after AI (*n* = 9; two maiden and seven multiparous; mares aged from 5 to 10 years; 7.78 ± 1.90);
2. Group 2. In this group, no fluid was detected in the uterus during estrus, but, 7 h after AI, more than 2 cm depth of fluid was detected in the uterus (*n* = 8; one maiden and seven multiparous; mares aged from 4 to19 years; 12.36 ± 5.68);
3. Group 3. Mares were enrolled into this group if more than 2 cm depth of IUF was detected in two consecutive examinations during estrus, and also 7h after AI (*n* = 8; one primiparous and seven multiparous; mares aged from 5 to 20 years; 15.33 ± 4.47).

### 2.4. Artificial Insemination

All mares were treated *i.v.* with 1500 IU of hCG (Chorulon^®^, Intervet, Holland) during estrus when endometrial edema and a dominant follicle (4 cm in diameter) were detected. Mares were inseminated 24 h after hCG treatment with commercially available semen shipped from different European centers from stallions with proven fertility. Prior to AI, the mare’s tail was bandaged, and the vulva and perineum were scrubbed with povidone-iodine and dried with a paper towel. An insemination dose (10 mL of chilled semen for each mare, with total number of sperm above 600 × 10^6^, and with spermatozoa motility above 75%) was deposited into the uterine body using a sterile insemination catheter inserted through the vagina and cervix by a hand covered with a sterile obstetrical sleeve. No treatments were applied before or after AI except sample collection from the uterus performed during estrus (at the time of hCG administration) and 7 h after AI, as described below.

### 2.5. Sample Collection

Fluid samples from the uterus were collected during estrus (endometrial edema and a dominant follicle close to but not exceeding 4 cm in diameter were detected) and again 7 h after AI. Prior to sample collection, the perineal region of the mare was prepared in the same way as for AI. Then, uterine lavage (UL) was performed using a commercial, sterile uterus flushing tube (EQUIVET Uterine Flushing Tube Sterile; Kruuse, Denmark) [4,27,34]. Briefly, the sterile catheter for UL was manually passed into the uterus by a hand covered with a sterile obstetrical sleeve and 60 mL of sterile physiological saline warmed to 38 °C was infused from a syringe into the uterus. The fluid was allowed to become distributed within the uterus while transrectal uterine massage was performed for about 1 min, and then the fluid was collected into sterile 50 mL conical tubes by gravity flow. Usually, more than 80% of the volume of the inserted fluid was recovered in mares without the presence of IUF in the uterus. In mares with the presence of IUF, more than 50 mL of the fluid was recovered. The fluid obtained was immediately centrifuged at 400× *g* for 10 min. The supernatant was transferred to 2-mL tubes and stored at −80 °C until cytokine and APP analysis was performed, and then a sterile swab was inserted into the sediment and used for cytological and microbiological examination.

### 2.6. Cytology and Microbiology

A swab with cellular material was gently rolled on a glass slide, which was dried and stained using Diff-Quick (Medion Diagnostics AG, Düdingen; Switzerland), and then evaluated by light microscopy. In order to evaluate inflammation, a total of 300 cells were counted under 400x magnification and the percentage of PMNs was recorded. When PMN cells represented more than 2% of all cells in the sample, it was considered positive for endometritis [34,35,36]. Microbiological examination was performed from material collected before AI as already described [27]. Bacterial cultures were quantified as follows: no growth, sparse growth (up to 10 colonies), moderate growth (11–50 colonies) or abundant growth (more than 50 colonies). The presence of infection was presumed if pure cultures of bacteria (>90% of the cultured colonies were of one species) were accompanied by positive results of cytology. Mixed growth of more than three microorganisms was considered as contamination.

### 2.7. Measurements of Cytokines

The concentrations of cytokines in uterine flushing samples were determined using commercially available enzyme-linked immunosorbent assays for measuring equine IL-1RA and IL-10 using the Thermo Scientific^TM^ Pierce^TM^ (Carlsbad, CA, USA) Equine IL-1RA (IL1RN) ELISA Kit and the Thermo Scientific™ Pierce™ (USA) Equine IL-10 ELISA Kit, respectively. All samples were analyzed in duplicate and run in accordance with the manufacturer’s instructions. The intra- and inter-assay coefficients of variation (CV) for all examined cytokines were <10% and <12%, respectively. The absorbance was read at a wavelength of 450 nm using 630 nm as a reference with a Biotek μQuant reader (BioTek Instruments Inc., Winooski, VT, USA).

### 2.8. Measurement of APPs

The concentrations of SAA were assessed using the Multispecies SAA ELISA Kit (Tridelta Development, TP 802, Maynooth, Ireland) according to the manufacturer’s instructions. All samples were analyzed in duplicate. The absorbance was read at a wavelength of 450 nm using 630 nm as a reference with a Biotek μQuant reader (BioTek Instruments Inc., Winooski, VT, USA). The intra- and inter-assay CVs were 2.1% and 5.2%, respectively. The concentration of Hp was determined by the guaiacol method according to Jones and Mould [37] and Kątnik et al. [38]. The intra-assay CV was 10.4% and the inter-assay CV was 13.5%.

### 2.9. Statistical Analysis

Variables for IL-1RA, IL-10, SAA and Hp were distributed according to the Shapiro–Wilk test. Due to the lack of normal distribution, the average values between Groups 1, 2 and 3 were compared using the Kruskal–Wallis test as a non-parametric alternative for the analysis of variance. Then, a series of post-hoc cross-tests were performed to determine their statistical significance. An analysis was also carried out in which the variables before and after insemination were compared within groups. For this purpose, the Wilcoxon matched pairs test was used. Data of a nominal nature (cytology before insemination, cytology after insemination, pregnancy results) were subjected to study the dependence on the variable group by Fisher’s exact test. The analysis was performed at the 5% significance level using STATISTICA statistical data packages, version 12 (StatSoft, Inc., Round Rock, TX, USA).

## 3. Results

These results were presented in preliminary form at the Japanese–Polish Joint Seminar in Warsaw, Poland, on 8–11 September, 2019 [39]. Mares from Group 1 were significantly younger compared to Group 3 (*p* = 0.008). The pregnancy rates were 66.67% (6/9), 62.5% (5/8) and 25.0% (2/8) in Groups 1, 2 and 3, respectively. The pregnancy results were not significantly different. The results of pregnancy rates did not change during second pregnancy diagnosis and after parturition. However, in Group 3, one foal was born a weak after prolonged gestation and died several days after delivery.

Microbiological examination revealed abundant (in one mare from Group 3) or moderate (in two mares from Group 3) growth of *Escherichia coli*, as well as sparse growth of various bacteria (β-hemolytic streptococci, coagulase-negative staphylococci or micrococci) in nine mares. However, none of these animals showed signs of inflammation upon cytology before AI. In the remaining 13 cases, no growth of bacteria was detected.

**Figure 1:**
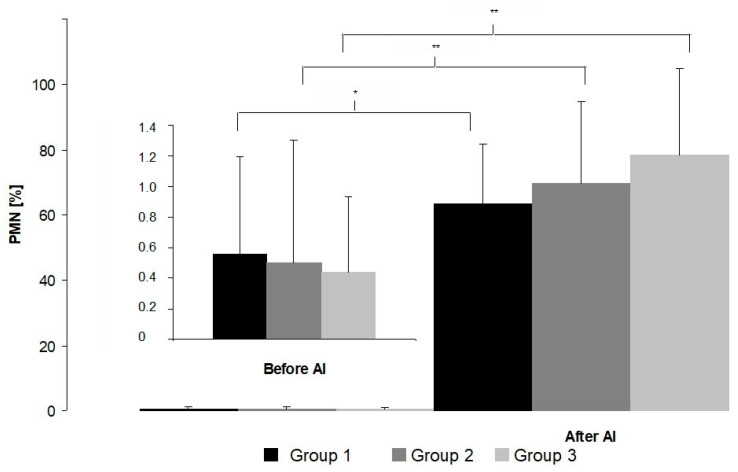
Percentage of polymorphonuclear (PMN) cells in cytological smears before and after artificial insemination (AI) (mean ± SD). Statistical significance set at * *p* = 0.008; ** *p* = 0.012.

In all groups of mares 7 h after AI, a significant increase in PMN cells was recorded (Figure 1). However, no differences were found among groups of mares before and after AI.

**Figure 2:**
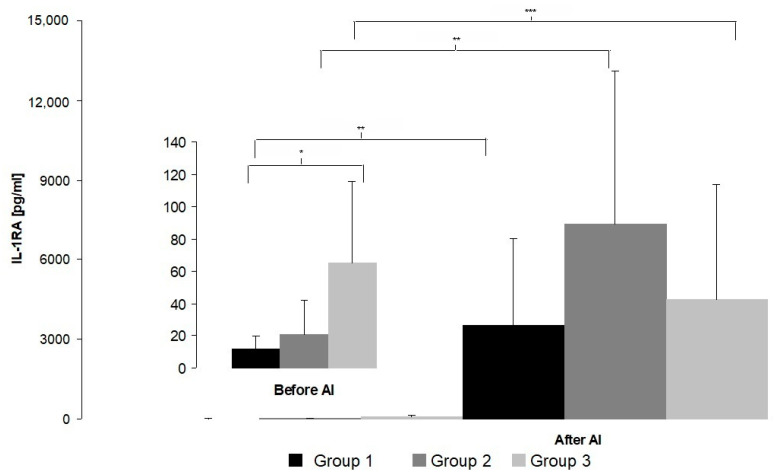
Concentration of IL-1RA before and after AI (mean ± SD). Statistical significance set at * *p* = 0.01; ** *p* = 0.008; *** *p* = 0.012.

The concentration of IL-1RA was significantly higher before AI in mares of Group 3 compared to those of Group 1 (Figure 2).

After AI, a significant increase in IL-1RA was observed in all three groups of mares. However, there were no significant differences among the groups of mares at this time.

**Figure 3:**
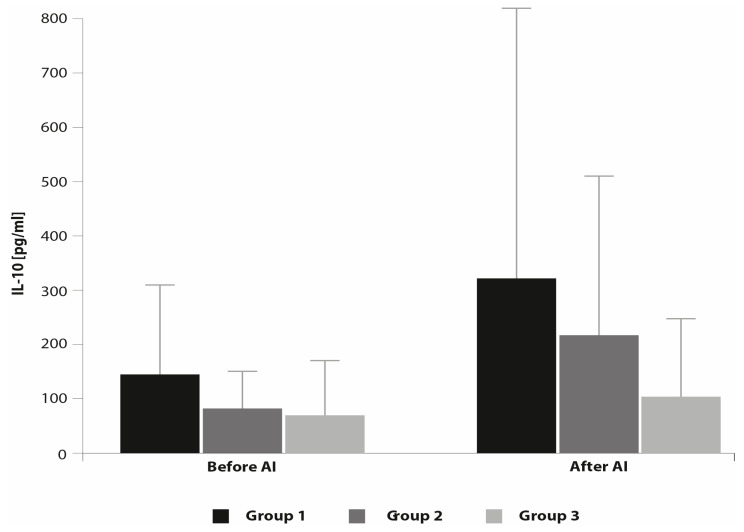
Concentration of IL-10 before and after AI (mean ± SD).

No significant differences were observed in the concentration of IL-10 among the groups of mares before and after AI (Figure 3).

**Figure 4:**
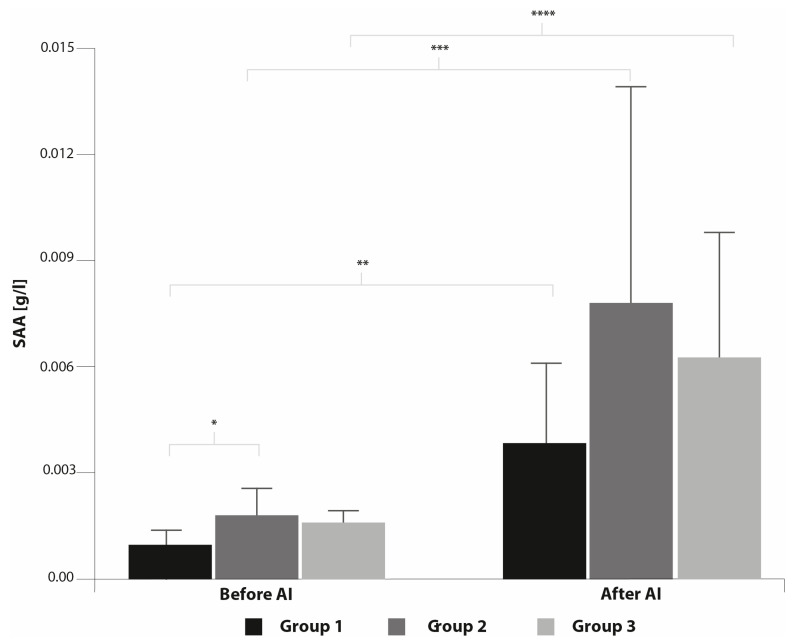
Concentration of serum amyloid A (SAA) before and after AI (mean ± SD). Statistical significance was set at * *p* = 0.041; ** *p =* 0.012; *** *p =* 0.017; **** *p* = 0.036.

The concentration of SAA before AI was significantly lower in Group 1 compared to Group 2 (Figure 4). In all groups of mares 7 h after AI, a significant increase in SAA concentration was recorded. However, no differences were found among the groups of mares after AI.

**Figure 5:**
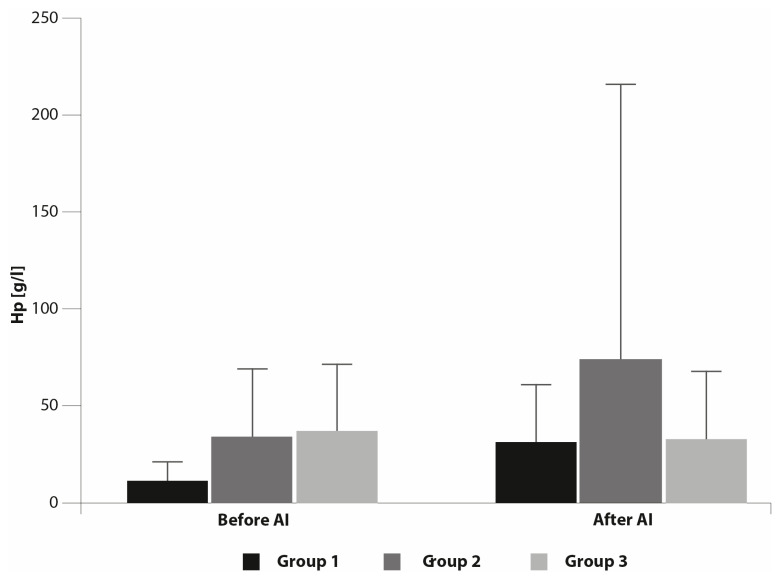
Concentration of haptoglobin (Hp) before and after AI (mean ± SD).

The results of measuring the concentration of Hp are shown in Figure 5. No significant differences were observed among the groups of mares before and after AI.

## 4. Discussion

The obtained results indicate that the presence of more than 2 cm of fluid in the uterus 7 h after AI does not affect fertility because the pregnancy rate in Group 2 can be considered high and was close to the results obtained in Group 1. In Group 2, clearing of the uterus from IUF was delayed compared with Group 1. Nevertheless, in most mares from this group, the uterine environment eventually was adequate for early pregnancy development, i.e., clearing of the uterus was completed before the embryo entered from the oviduct. In Group 3, only two mares were pregnant. It should be pointed out that this group of mares was the oldest, and the age could affect the fertility rates. Additionally, in three mares from Group 3 *E. coli* was isolated from flushes obtained before AI, but results of cytology were negative. LeBlanc et al. [34] considered that the flushing should be regarded as contaminated if bacteria are isolated but the efflux is clear, there is no change in pH, cytology is either hypocellular or non-inflammatory, and, if biopsy is obtained, no neutrophils are present in the endometrial tissue.

In normal mares, the first PMN cells are detected in the uterus 0.5 h after AI, reaching a peak at 8h and remaining at high levels until 24 h, and then almost completely disappear 48 h after AI [2]. In our study, in all groups of mares, a significant increase in PMN cells was observed after AI. However, no significant differences were observed among the groups of mares before or after AI. The lack of differences in the numbers of PMN before AI among all groups supports previous observations that the presence of fluid in the uterus during estrus may not be conclusive evidence of inflammation [15,16]. After AI, we found no significant differences in the numbers of PMN among the three groups of mares. Our results are in agreement with findings obtained by Woodward et al. [22], where, 6 h after breeding, the numbers of PMN increased in both mares that were susceptible and resistant to PBIE. Analyzing the profiles of PMN numbers after insemination, Nash et al. [4] concluded that PMN numbers that remain elevated at 16 h after challenge may be indicative of susceptibility to PBIE.

After AI, not only did the number of PMN cells increase, but also SAA, another marker of inflammation, significantly increased after AI in all groups of mares. Generally, APP production in the liver is stimulated by pro-inflammatory cytokine IL-6 which originates from the inflammatory site and then other cytokines may influence the pattern of acute-phase response proteins induced by IL-6 [40]. Nevertheless, gene expression for SAA was found in the mare endometrium [22] and changes in APP concentrations were observed in uterine flushings collected from cows suffering from endometritis [31,32]. The precise function of SAA is not fully understood but among others it can also stimulate PMN migration [41]. This explains the elevated concentrations of SAA in uterine flushings and simultaneous PMN migration into the uterine lumen in response to AI. However, no changes were observed in plasma concentrations of SAA and other APPs in response to AI in horses [4,29]. Furthermore, it was shown that plasma concentrations of SAA and Hp are not useful tools for diagnosing subclinical endometritis in mares [27].

We observed that SAA concentration was significantly lower before AI in Group 1 compared with Group 2 mares but no differences were detected in comparison with Group 3. In Group 2, one mare, for unknown reasons, had at this time an elevated concentration of SAA. Christoffersen et al. [23] reported no differences at estrus in gene expression levels of SAA between resistant and susceptible mares. Additionally, Christoffersen et al. [23] observed no significant difference in endometrial mRNA transcripts for SAA between resistant and susceptible mares after intrauterine infusion of *E. coli*.

We did not observe significant changes in Hp concentrations in uterine flushings collected before and after AI. In contrast to SAA, which is the major APP, and a rapidly responding and very sensitive marker of inflammation, Hp is a minor-to-moderate APP and its increase is slower and characteristic of chronic inflammation [42,43].

We chose the time point of sample collection at 7 h after AI based on the work by Woodward et al. [22]. This is because differences detected in cytokine mRNA expression 6 h after challenge between mares susceptible or resistant to PBIE can suggest that time spent on the establishment of the mechanism for uterine response and clearance is critical. In our study, we measured products of gene expression, i.e., cytokine protein concentrations in uterine lavage fluids, and it should be pointed out that data from the literature show that correlations of mRNA and protein levels are frequently far from perfect [44,45]. Such differences were also observed in mare endometrial tissue when mRNA expression and protein concentrations for some cytokines were measured [46,47]. The background of poor correlations between mRNA expression levels and protein concentrations can be explained by the existence of complex mechanisms involved in post-transcriptional and post-translational processes; additionally, experimental error cannot be excluded [44,45].

We observed no significant differences in the concentration of anti-inflammatory IL-1RA and IL-10 measured 7 h after AI among all the groups of mares. Woodward et al. [22] showed that 6h after AI, mRNA expression of IL-6, IL1RN and IL-10 was higher in resistant mares than in those susceptible to PBIE, and they concluded that around 6h after insemination may be a critical time in the establishment of the mechanism for uterine response and clearance. Palm et al. [48] reported increased gene expression of pro-inflammatory cytokines IL-1β, IL-6 and TNFα in reproductively normal pony mares 12 h after infusion of PBS, seminal plasma or semen extender into the uterus. It appears that the response of the endometrium after challenge is time-dependent and can depend on the irritating agent. Additionally, the health status of the endometrium and the severity of inflammation impacts the immune response and fosters activation of innate immunity mechanisms [46]. In the study by Reilas et al. [49] aimed at the effects of cervical occlusion on the equine endometrium, no changes were observed in the presence of cytokine (IL-1β, IL-6, IL-10 and TNF-α) proteins in uterine fluid 25 h after AI. However, the results are not totally comparable since, in the study from Reilas et al. [49], cytokines were evaluated by Western blotting and not by ELISA. Fumuso et al. [19] reported that differences in the endometrial expression of mRNA for IL-1β, IL-6 and TNFα 24 h after AI were not significantly different between mares that were susceptible and resistant to PBIE. However, mRNA expression for all three pro-inflammatory cytokines increased 24 h after AI compared with baseline expression in both groups of mares, but differences were only significant in the group of resistant mares. In another study, there was no change in the expression of IL-8 24 h after AI in normal pony mares but concentrations of PGF_2_α measured in uterine lavage fluid significantly increased 16 h after challenge and returned towards basal levels by 72 h [4]. Based on this, it is probably too late to find any differences in cytokine production or their gene expression levels a day after challenge.

We observed that IL-1RA concentrations were significantly higher before AI in Group 3 compared with Group 1 mares. At this time, we observed no differences between these two groups in cytological examination results. It is widely accepted that the presence of IUF during estrus is usually not connected with inflammation manifested by the recruitment of PMN cells, but in these mares elevated concentrations of anti-inflammatory cytokines suggests that the presence of fluid induces an anti-inflammatory response. Christoffersen et al. [23] reported no differences at estrus in gene expression levels of any cytokines studied between resistant and susceptible mares, but Fumoso et al. [19] showed that endometrial mRNA expression for pro-inflammatory cytokines was significantly higher during estrus in mares susceptible vs. resistant to PBIE.

It is worth noting that our study has some limitations. We are aware of the lack of a control group in which, instead of semen, saline solution would have been infused into the uterus. Moreover, the collection of endometrial biopsies and the direct analysis of cytokines and APP in the endometrial tissue would have also added more power to the study.

## 5. Conclusions

Seven hours after AI, regardless of the status of the mare before AI, the endometrial response is characterized by PMN influx, while SAA, Hp, IL-1RA and IL-10 production is similar. The status of the mare before AI has little effect on the response measured 7 h after AI. The presence of IUF during estrus is not related to PMN influx but can impact uterine IL-1RA production at this time.
